# A first report of East Asian students’ perception of progress testing: a focus group study

**DOI:** 10.1186/s12909-016-0766-2

**Published:** 2016-09-22

**Authors:** Yasushi Matsuyama, Arno M. M. Muijtjens, Makoto Kikukawa, Renee Stalmeijer, Reiko Murakami, Shizukiyo Ishikawa, Hitoaki Okazaki

**Affiliations:** 1 Medical Education Center, Jichi Medical University, 3311-1 Yakushiji, Shimotsuke, 329-0498 Tochigi Japan; 2 Department of Educational Development and Research, Faculty of Health, Medicine, and Life Sciences, Maastricht University, 6200 MD Maastricht, The Netherlands; 3 Department of Medical Education, Kyushu University, 3-1-1 Maidashi, Higashi-ku, Fukuoka, Kyushu Japan; 4 Jichi Medical University of Nursing, Japan, 3311-1 Yakushiji, Shimotsuke, Tochigi Japan

**Keywords:** Assessment, Progress testing, Applicability, Undergraduate education, Japan, East Asia, Focus group

## Abstract

**Background:**

Progress testing (PT) is used in Western countries to evaluate students’ level of functional knowledge, and to enhance meaning-oriented and self-directed learning. However, the use of PT has not been investigated in East Asia, where reproduction-oriented and teacher-centered learning styles prevail. Here, we explored the applicability of PT by focusing on student perceptions.

**Methods:**

Twenty-four students from Years 2, 3, and 5 at Jichi Medical University in Japan attended a pilot PT session preceded by a brief introduction of its concept and procedures. Variations in obtained test scores were analyzed by year, and student perceptions of PT were explored using focus groups.

**Results:**

Formula scores (mean ± standard deviation) in Years 2, 3, and 5 were 12.63 ± 3.53, 35.88 ± 14.53, and 71.00 ± 18.31, respectively. Qualitative descriptive analysis of focus group data showed that students disfavored testing of medical knowledge without tangible goals, but instead favored repetitive assessment of knowledge that had been learned and was tested on a unit basis in the past in order to achieve deep learning. Further, students of all school years considered that post-test explanatory lectures by teachers were necessary.

**Conclusions:**

East Asian students’ perceptions indicated that, in addition to their intensive memorization within narrow test domains compartmentalized by end-of-unit tests, the concept of PT was suitable for repetitive memorization, as it helped them to integrate their knowledge and to increase their understanding. Post-test explanatory lectures might lessen their dislike of the intangible goals of PT, but at the expense of delaying the development of self-directed learning. Key issues for the optimization of PT in East Asia may include administration of PT after completed end-of-unit tests and a gradual change in feedback methodology over school years from test-oriented post-test lectures to the provision of literature references only, as a means of enhancing test self-review and self-directed learning.

**Electronic supplementary material:**

The online version of this article (doi:10.1186/s12909-016-0766-2) contains supplementary material, which is available to authorized users.

## Background

Agreement on optimum assessment procedures among students and teaching staff is an important element in maintaining the quality of assessment [1]. Van der Vleuten [2] stated that “a reliable, valid, and feasible test will have a short life if it’s accepted by no one.” When introducing new test procedures, it is critical to evaluate their acceptability among stakeholders—including students themselves. Student perception should be thoroughly explored before official implementation [1, 2].

Progress testing (PT) is a longitudinal testing approach developed in the USA and the Netherlands more than 30 years ago. The complete domain of knowledge considered essential for medical students by the time of graduation is periodically (i.e. 4 times a year in the Netherlands) tested throughout all curriculum years [3–5]. Test items are drawn randomly from a large bank of multiple choice or true/false questions. As most questions require students to integrate functional knowledge of different disciplines taught throughout the curriculum, examinees of PT cannot rely solely on rote knowledge, contrary to summative end-of-unit tests. In turn, students are motivated to study constantly to increase their functional knowledge in a self-directed manner. Additionally, frequent and repeated examinations requiring integrated knowledge are considered to promote better retention of meaningful information [3–8]. A few reports have been published regarding student perception of PT. Year 2 undergraduate students in the UK reported that constant learning, intrinsic motivation, and understanding and application of knowledge to clinical settings were important for successful PT [9]. Students in the Netherlands felt that, rather than being reproduction-oriented, PT is a meaning-oriented assessment [10].

However, the acceptability of novel education procedures is not always consistent across different cultures. For example, East Asian students preferred explanatory lectures by a teacher to lessen anxiety in problem-based learning (PBL) discussion while “full” PBL implementation encouraged students in the Middle East and the Netherland to engage in more critical discussions in PBL [11].

In East Asia, which is often defined as “China and the countries that were heavily influenced by its culture, most notably Japan and Korea [12]”, virtue is achieved primarily by learning from teachers and imitating their attitudes [13]. Teachers are placed in a superior position, and in return, students highly expect teachers to provide high-quality education and possess a positive personality [14, 15]. School examinations tend to emphasize accuracy in the reproduction of information, which teachers give in lectures and write in syllabus [14–16].

China, Japan, and Korea are regarded as having a similar attitudes and behaviors toward examinations. These are referred to as an *exam-oriented mindset* or *examination superstition* [14–16]. This mindset develops in the preuniversity phase, when students are asked by family members, teachers, and society to obtain high grade points and high rankings, because these will enable them to attend prestigious universities, and consequently assure their future success [15]. Preparatory cram schools play an important role in university entrance success. Tutors in cram schools devise strategies to repetitively review past lessons (such as past examination papers) and students try to memorize and reproduce what tutors teach as a means of preparing for entrance examinations [16]. Accordingly, it is considered that students at matriculation tend to rely on rote memorization to reproduce statements made by teachers when they take assessment tests [14, 15].

Given these cultural characteristics, East Asian students may encounter issues with accepting PT, as it will require that they abandon their reproduction-oriented, teacher-centered learning style and adapt instead to a style that is meaning-oriented and self-directed. To date, however, no study has reported the use of PT in East Asia, despite the increasing popularity of PT in Africa, South America, and other nations worldwide [3, 5–8, 17, 18].

Here, we evaluated student perceptions of PT in an East Asian population. We were particularly interested in determining students’ ability to recognize the advantages of PT at the point of introduction, and how students adjust their learning styles.

## Methods

### Setting

We elected to restrict this study to Japan to ensure a homogeneous focus group that consisted of Japanese students only. In this way, we could ensure the lack of any language barrier that would hamper productive discussion among participants and appropriate interpretation by Japanese researchers. We hoped that this homogeneity would maximize the advantages of focus groups, such as their enrichment of interviewee expressions and the exchange of information on subjects of mutual interest and concern [19]. We also assumed that, given that all focus group participants were from the same university, they would be more likely to actively participate in the discussion and disclose their own values or norms in learning than unacquainted participants from different universities. For these reasons, we chose a single medical university (Jichi Medical University; JMU) in Japan as the study setting.

The curriculum of JMU complies with the standardized model core curriculum, which outlines fundamental learning contents for undergraduate medical education in Japan [20]. While the curriculum is integrated in part, it remains mostly stepwise: during preclinical education before their clinical clerkship, students mainly learn clinical medicine in traditional didactic lectures, and their progress is assessed through end-of-unit tests. Given these characteristics [14, 15, 20, 21], we consider that the JMU curriculum remains traditional, making JMU an appropriate setting for this study.

Details of the JMU curriculum are described in Table 1. JMU students study liberal arts in the first and second trimesters of Year 1. Lectures and experiments in basic medicine also begin in the second trimester of Year 1. Lectures on clinical medicine start from the second trimester of Year 2. Each basic and clinical medicine class is capped by end-of-unit tests given in non-standardized test formats, where lecturers create test items based on what they taught. Before the end of Year 3, students finish lectures on almost every subject in basic and clinical medicine, with the exception of palliative medicine and clinical pharmacology. From Year 4 to the first trimester of Year 6, students are permitted to participate in clinical clerkship, during which they will take part in medical practice and receive training centered mainly on taking patient histories and providing physical examinations. From the first trimester of Year 6, graduation tests for each clinical subject commence; students must pass every test to graduate, and the test formats differ by subject. In addition to these graduation tests for each subject, students must pass a yearly comprehensive assessment test (the “Sougou-hantei Shiken” in Japanese) mimicking the national licensing examination, which covers comprehensive knowledge on both basic and clinical medicine at the end of Year 4 to 6. The exam coverage of the Sougou-hantei Shiken becomes broader as the school year increases. After passing these exams, students are allowed to graduate and apply for the national licensing exam. The national licensing exam consists only of a paper-based test with 500 multiple-choice questions and no practical skill examination. The examination pass rate averages approximately 90 % [20].

**Table 1:** Undergraduate curriculum at Jichi Medical University

Trimester	Year 1	Year 2	Year 3	Year 4	Year 5	Year 6
First (April to July)	Liberal arts	Basic medicine	Basic medicine/Clinical medicine	Clinical clerk-ship	Clinical clerk-ship	Clinical clerkship/Graduation tests
Second (September to December)	Liberal arts/Basic medicine	Basic medicine/Clinical medicine	Basic medicine/Clinical medicine			Graduation tests/Year 6 Sougou-hantei Shiken
Third (January to March)	Basic medicine	Basic medicine/Clinical medicine	Basic medicine/ Clinical medicine	Year4 Sougou-hantei Shiken	Year 5 Sougou-hantei Shiken	
			Preclinical CBT/OSCE			National licensing exam

*Abbreviations*: *CBT* computer-based test, *OSCE* objective structured clinical examination

The transferability of findings in this study to East Asian countries can be assured based on the commonality in cultural values [14, 15] and learning styles in the preuniversity phase [15, 16], the similarity of traditional undergraduate curricula, and the existence of the national licensing exams as an absolute prerequisite to becoming medical doctors [20–23].

### Subjects

To capture a variety of perceptions across years of education, we randomly selected eight students (four men, four women) from the JMU student roster for each of Years 2, 3, and 5 (*n* = 24 total). We chose students from these years because student from Years 1, 4, and 6 were unable to participate due to test scheduling conflicts.

Instead of purposeful sampling, which is often used in qualitative studies to select participants [24], we used randomization to choose potential participants because the ethics board warned that purposeful sampling might cause researchers to access individual students’ information before the initial agreement and pressure them to participate.

In detail, we held a 30-min explanatory session about the concept and rules of PT and the purpose of this study for almost all Year 2, 3, and 5 students (approximately 110 in each year group) 1 month before the start of this study. We also informed them that eight students would be randomly chosen from each school year group and would receive an e-mail invitation to the study, asking whether they would be able to enroll in the study or not. After the student’s agreement via e-mail, researchers made the first contact with participants and obtained their written consent for research and publication.

Only five students in total did not respond to the email, and were replaced by additional randomly selected students. No participant spontaneously applied to participate in this study. Therefore, we could see that the participants were not those from biased populations.

### Design

The study was conducted from October 2014 to March 2015 and comprised four parts: 1) construction and implementation of a pilot progress test, 2) analysis of test scores, 3) organization of focus groups, and 4) qualitative descriptive analysis of the focus group data.

1. Construction and implementation of a progress test
  A 2-h pilot progress test with 100 multiple-choice questions was created for this study, covering basic and clinical medicine in the areas of general internal medicine, cardiology, pulmonology, gastroenterology, hematology, nephrology, endocrinology, neurology, and rheumatology. All test items were thoroughly checked by members of the Medical Education Center at JMU.
  The pilot progress test was simultaneously administered to all participants (*N* = 24) in a single room. Before taking the test, participants attended a 30-min explanatory session on PT to aid in their recall of the concept and rules by referring to the current operational conditions of the Dutch consortium (4 test administrations per year, 200 multiple choice questions per test, nationwide multicenter administration, feedback information provided to students after the test, etc.) [5, 25]. In addition, it was explained that total annual scores would become part of the criteria for advancing to the next grade-level/graduation, and that PT is being used along with other summative/formative assessments in Western countries. Students were also informed that PT used multiple-choice questions with a “don’t know” option that they could choose when a question covered material not yet taught or far beyond the student’s academic level. They were told that this option could help them avoid incurring a penalty (minus one divided by the number of distractors) for an incorrect answer. This followed the rule of the Dutch consortium [5]. In this trial test, each item had one correct answer and four distractors; therefore, the penalty for a wrong answer was minus 0.25. They were also informed that the researchers were trying to determine how best to administer PT and welcomed any ideas on adapting PT to the Japanese learning environment.
  After the test, each participant received a handout showing the correct answers along with explanations and literature references for each question. The students were permitted to score and review the test by themselves and were allowed to ask questions of teachers participating in the study.
2. Analysis of test scores
  For each school-year group, we calculated the mean and standard deviation (SD) of the formula score (the number of correct items −0.25 × the number of incorrect items, expressed as a percentage of the number of items in the test), the percentage of correct answers, the percentage of incorrect answers, and the percentage of “don’t know” responses.
  Differences in test scores between Years 2, 3, and 5 were statistically analyzed using one-way ANOVA. A *p*-value < 0.05 was considered statistically significant. When ANOVA showed statistically significant differences, the effect sizes (Cohen’s *d*) for comparisons between Years 2 and 3, Years 2 and 5, and Years 3 and 5 were calculated. Cohen’s *d* values of 0.2, 0.5, and 0.8 were considered to indicate differences of negligible, moderate, and crucial practical importance, respectively [26].
3. Focus groups
  Focus groups are an effective way of obtaining qualitative data from discussions when the degree of familiarity with the topic is uniform and when the power relations between discussants are weak and they feel comfortable sharing experiences and exchanging ideas [19, 24]. In the present study, we assumed that focus groups comprising eight same-year students from the same school would facilitate the sharing of ideas and concerns with minimal hesitation.
  Six days after the test, all participants attended the 60-min focus group session, except for one Year 3 male student (no reason provided for absence). Participants were divided into groups by school year, and each group had one moderator (a medical doctor within the department of gastroenterology who did not take part in this study as a researcher). The moderator was informed of his role in accordance with the AMEE guide for focus groups [19] and had taken part in one trial session with simulated participants (staff members) beforehand.
  All conversation during the session were recorded and transcribed by three research assistants. Participants were not identified in order to guarantee anonymity.
4. Descriptive qualitative analysis
  The transcribed anonymous scripts were analyzed by the main researcher (YM) and one medical educator (MK), who had a neutral opinion regarding PT. The analysis was conducted in line with descriptive qualitative analysis [27]. The transcripts were thoroughly read and analyzed using an inductive coding approach. Words and phrases extracted from the transcripts were first individually coded by the two researchers (YM and MK), and agreement on coding was achieved through Skype meetings between the pair. These two researchers initially identified 20 codes, which were listed by school year. All students were invited to confirm whether or not these codes appropriately captured their perceptions (member checking); no protests were lodged regarding any part of the content at this point. After coding confirmation, the other authors (RS, AM, SI, and HO) joined the discussion, and a higher level of synthesis of the codes was obtained, resulting in three major themes.

## Results

### Test results

**Fig. 1:**
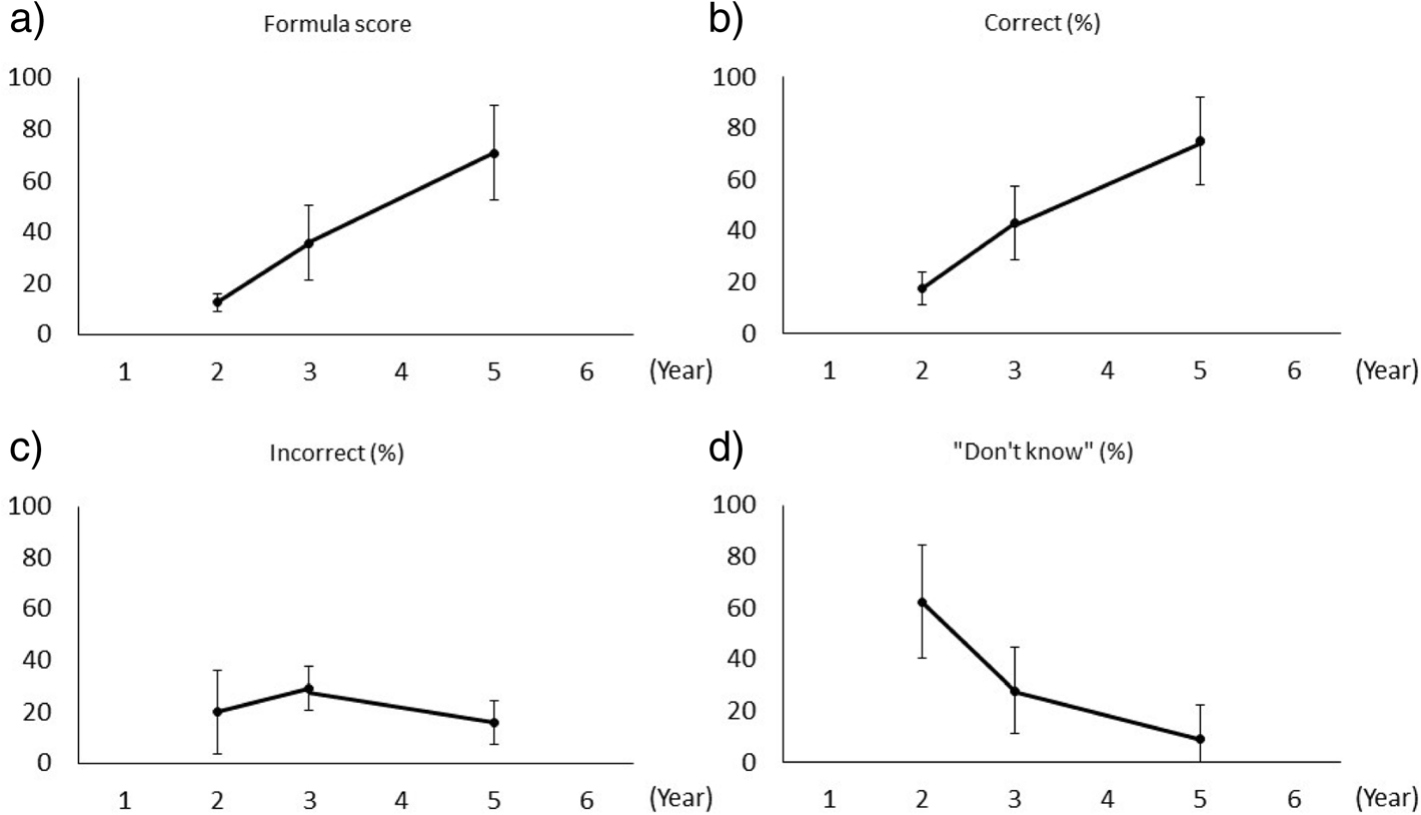
Mean scores of the trial test. Scores for the 3 samples of 8 students of Years 2, 3, and 5 (X-axis) that attended the trial progress test. The Y-axis indicates: **a** percentage correct minus penalty for incorrect answers, **b** percentage correct, **c** percentage incorrect, and **d** percentage of “don’t know” responses. The point on the line indicates the mean score of the year group, and the vertical bar indicates the standard deviation. One-way ANOVA showed significant differences between Years 2, 3, and 5 for scores **a**), **b**), and **d**)

Student formula score and percentage correct increased linearly with school year (Fig. 1). Formula scores (mean ± SD) for Years 2, 3, and 5 were 12.63 ± 3.53, 35.88 ± 14.53, and 71.00 ± 18.31, respectively. The percentage of “don’t know” responses decreased with increasing school year. Between-year differences in formula score, percentage correct, and percentage of “don’t know” responses were found to be statistically significant using one-way ANOVA (F (2, 21) = 37.1, *p* < 0.001; F (2, 21) = 38.3, *p* < 0.001; F (2, 21) = 18.6, *p* < 0.001, respectively) and Cohen’s *d* value (Table 2). The average percentage of incorrect responses was less than 30 % across all years, with no statistical significance between years (F (2, 21) = 2.6, *p* = 0.098).

**Table 2:** Cohen’s *d* in formula score, percentage correct, and percentage of “don’t know” responses

Year	Formula score	Correct (%)	Don’t know (%)
2 vs. 3	2.20	2.29	1.77
3 vs. 5	2.13	2.03	1.25
2 vs. 5	4.43	4.48	2.92

### Focus groups and qualitative descriptive analysis

Analysis revealed three themes: participants (1) disfavored testing of medical knowledge without tangible goals, (2) favored repetitive assessment of knowledge learned in the past, and (3) felt that post-test explanatory lectures by teachers were necessary.

- Theme 1. Participants disfavored testing of medical knowledge without tangible goals.
  Overall, participants demonstrated their disfavor of PT by discussing the benefits of summative end-of-unit tests of each subject. They felt that end-of-unit tests showed “attainable goals” and helped them focus on what they were supposed to study, enabling them to spend their energy and time reading and understanding what they should learn from the textbook and teachers’ statements.
  *“Attainable goals…the conventional end-of-unit tests that are administered frequently have a clear goal for which I’m driven to study hard.”* (Year 2, male)
  *“We cannot answer the current (end-of-unit) tests unless we thoroughly read textbooks and understand the pathophysiology.”* (Year 3, male)
  Participants felt that intensive studying within a narrow domain in preparation for end-of-unit tests was important, as they believed that the information learned before the test could then be easily retrieved from memory later in their curriculum.
  *“The fact that I experienced the (end-of-unit) test is important… The knowledge I once learned in the past was easily regained, even though we tend to forget it soon after (the test).”* (Year 3, male)
  On discussing how they might feel switching from end-of-unit testing to progress testing, participants articulated their feelings with the word “confusion” in regard to the distance between their final achievement goals and their current studies.
  *“It was too difficult to identify the distance between where I am now and where I will be at the final goal…. As a result, that would enhance confusion.”* (Year 2, male)
  Their devastating “confusion” status was exemplified by remarks of some Year 2 students. During the pilot test, seeing the many “don’t know” options they had marked, made them feel disconcerted and spiritless. After the test, the “don’t know” marked questions did not prompt them to study in order to fill in the gap between their final achievement goals and their current understanding; instead the gap just made them feel disappointed and spiritless.
  *“When I saw tremendous “don’t know” marks, I became emotionally distressed…then I fell asleep for the final hour of the test.”* (Year 2, male)
  *“Many would terminate the self-review, saying “wow…it cannot be done anyhow.”* (Year 2, male)
  Participants then went on to explain that, in order to ameliorate this “confusion”, the PT should begin no earlier than the second trimester of Year 2, when most clinical subjects of medicine begin. They felt that Year 4 and 5 students, who have finished nearly all lecture courses and started their clinical clerkships, would be the best candidates for PT.
  *“After the second trimester of Year 2 or Year 3, lectures of (most clinical subjects of) medicine will start. Then, we expect that we will see a “development period” through the progress test.”* (Year 2, male)
  *“I think that Year 4 and 5 students might benefit the most from progress testing.”* (Year 5, male)
- Theme 2. Participants favored repetitive assessment of knowledge learned in the past
  When participants were questioned about what they liked about PT, Years 3 and 5 students (who have completed end-of-unit tests for almost all clinical subjects) stated that PT gave them repeated opportunities to integrate knowledge in a specific domain that had already been learned and tested in end-of-unit tests, which would subsequently strengthen their understanding of this knowledge.
  *“Because we’ve already been taught all of these subjects, we were able to link them together while answering the questions.”* (Year 3, female)
  “*If we are repeatedly tested on the same information, I can confirm my knowledge, and I will gradually come to understand its importance to my education.”* (Year 3, female)
  They recognized that repetitive memorization through PT would help not only strengthen their knowledge but also shift them from a passive learning style of rote memorization to an active and constant learning style.
  *“I have been passively taught by lecturers. I listened to them and said only ‘I see, I see’… I need to actively output the knowledge that I absorbed when listing differential diagnoses… It’s the best way to study… I expect the progress test to help in that respect.”* (Year 3, male)
  *“While I view rote learning unfavorably, I can’t stop it. In this sense, I’d say, PT is advantageous (in order to stop studying by one-shot rote memorization).”* (Year 5, female)
- Theme 3. Participants feel that post-test explanatory lectures by teachers are necessary.
  Students in all school years felt that explanatory post-test lectures were necessary, as they believed that post-test lectures presented an opportunity to review the test more attentively before teachers than self-learning and clarify their position in the learning process through the direct instruction of teachers.
  *“A situation that prompts me to study…may encourage me more. I want to secure enough time for explanatory lectures.”* (Year 3, female)
  *“Teachers could give lectures for each school year. They could also show data to help students understand their positions…and what they should study as an underclassman or an upperclassman…”* (Year 5, female)
  In response to their devastating “confusion” pertaining to the distance between the final achievement of goals and studies in the earlier school years (as described in theme 1), no participants mentioned that they would be encouraged to adjust their learning strategies by themselves. On the other hand, they strongly counted on post-test explanatory lectures to clarify the association between their final achievement goals and studies in the earlier school years, and to provide instruction to help shorten the distance between them.
  *“This is important, this is what you should learn, and so on. If they formulate such a concrete explanation which shows the track (to the goal), implementing it (PT) would become significant”* (Year 2, male)

## Discussion

In this study, we conducted a single pilot test following the form of PT in a medical university in East Asia. The student formula score and percentage of correct answers in the pilot test increased with school year while the percentage of “don’t know” responses decreased, findings similar to those observed in Western countries [7, 8, 25]. However, the similarity of the test results does not indicate that the introduction of PT in East Asia would be straightforward. After participating in the pilot test, homogenous focus groups with discussants of the same school year described their perceptions of the advantages and disadvantages of PT. These focus groups also gave students a platform for making suggestions on how to improve PT or how to adjust themselves to PT. To our knowledge, this is the first report pertaining to students’ perceptions toward PT in an East Asia context.

In the focus groups, the students elaborated on the unacceptable points of PT by discussing their current end-of-unit tests. They also described their learning preferences, and the advantages they saw with end-of-unit tests over PT. Preparation for end-of-unit tests plays a central role in their learning style, and students found it important to master the well-defined domain of knowledge presented in their textbooks and class lectures by reproducing them thoroughly and perfectly.

Kember [28] described a similar characteristic in the learning styles of other East Asian students that he referred to as the “narrow approach.” In this learning style, students work systematically section-by-section, mastering material before proceeding to the next section. We therefore speculate that East Asian students in the present study may have been confused by being tested on knowledge they had yet to encounter in their lectures or in summative end-of-unit tests. Given that students in the present study evaluated PT from the perspective of whether or not they could answer test questions using past knowledge, a lack of such knowledge may have biased opinions against PT. This remark is consistent with a discipline of Confucianism which states, “Study the past if you would define the future”. Given this, any testing style which covers a domain of information which must be learned at a future point in the curriculum might be unacceptable to East Asian students, regardless of its purported utility.

Our focus groups participants did not express clear opinions on how they could utilize progress tests in combination with end-of-unit tests in early school years. However, negative attitudes like sleeping during the pilot test and the abandonment of self-reviewing by most Year 2 participants suggest that it would not be advisable to introduce PT to students who have not completed end-of-unit tests for most subjects.

While PT might be in conflict with intensive rote memorization (or the “narrow approach”) during end-of-unit tests at the preclinical stage, our Year 3 and 5 students did recognize the utility of PT with respect to their preference for repetitive memorization. They appreciated PT as a learning opportunity, which might enhance repetitive memorization by helping them comprehend what they have learned through rote memorization in end-of-unit tests, and eventually acquire integrated comprehensive knowledge, a supposed prerequisite for being a safe and effective professional.

Purdie & Hattie [29] reported that Japanese high-school students preferred to use repetitive memorization as a route to meaning-oriented understanding by referring to the Confucian proverb, “Read it one hundred times and understanding will follow spontaneously.” Purdie & Hattie claimed that Japanese students utilize repetitive memorization as if they are making choices about learning contents. In other words, they choose what is necessary or unnecessary for their current knowledge while memorizing. They also discussed the strong exertion of willpower in learning, a notion which is congruent with the Japanese cultural emphasis on commitment to a task and the virtue of perseverance, and that such exertion might strengthen the advantages of repetitive memorization. Watkins [30] similarly identified a three-stage learning approach among senior high school students in Hong Kong. In the first stage, students attempt to master thorough reproduction of knowledge by rote-learning. However, as they advance through school and their memory load becomes saturated, they begin to distinguish importance, discerning what they should remember and what they may forget. Finally, students start to see the benefit of trying to understand material by themselves before committing it to memory.

Indeed, students in the present study who had already completed their summative end-of-unit tests for most basic and clinical medicine subjects stated that they felt PT would help them shift from passive learning via repetitive memorization to active and meaning-oriented learning. This shift in learning preference seems to correspond to the learning steps in Watkins’ theory [30].

Another aspect of the East Asian learning style is the strong demand for post-test lectures. Students felt the need for feedback, not only in the form of objective data but also in explanatory lectures from teachers practicing PT. Although Western students also perceived the importance of feedback in PT [9, 10, 31], subjects in these previous studies did not specify the desired route of receiving feedback. This contrasts with our present East Asian students, who emphasized the importance of explanatory lectures by teachers. Student preference for instruction from teachers is consistently observed in preuniversity settings in East Asia [15, 16]. We anticipated that earlier school year students, who had just passed competitive entrance exams with the help of lecturers at private cram schools, would be inclined to rely on instructional lectures. We therefore expected that lecture-based feedback would be an appropriate strategy to lessen confusion in earlier school years. However, we did not expect that Year 5 students, who were training in clinical clerkships, would also persistently rely on lectures and make little effort in applying themselves to the concept of PT. We were therefore surprised at this finding.

The delayed development of self-directness we experienced in our study groups is problematic, because deficits in self-directed learning, such as inadequate self-monitoring and insufficient self-reflection, are robust predictors of poor work performance [32, 33]. In medical practice, doctors cannot await the support of teachers. Students in later school years who have perceived the advantage of PT as an opportunity to strengthen their repetitive memorization and accept PT as an opportunity to become active and constant learners should therefore be provided PT to help them become accustomed to self-directed learning. A gradual decrease in lecture-based feedback and a gradual increase in self-review opportunities using handouts with references to relevant literature, such as the Dutch consortium provides [5, 25], could be implemented.

Overall, PT is considered beneficial for East Asian students as a means of helping strengthen their repetitive memorization after they have come to recognize the limitations of intensive rote memorization by the “narrow approach.” Feedback methods may be considered a key issue for optimizing PT in East Asia, and a shift from lecture-based feedback to self-reviewing might be optimal.

### Limitations and further research

Several limitations to the present study warrant mention.

- First, participants in the present study were informed that PT is being used along with other summative/formative assessments in Western countries. This information might have invited participants to imagine the setting of a hybrid PT model, in which summative end-of-unit tests are used along with PT.
- Second, our investigation in East Asian students employed a single pilot test only. We were therefore unable to explore student perceptions from the perspective of receiving PT several times a year, albeit the present study satisfied the initial curiosity of students’ perceptions of PT at the point of introduction. Further long-term research is needed to address this limitation.
- Third, the influence of the current curriculum itself cannot be ignored. Although the curriculum of undergraduate education in Japan is standardized in accordance with the model core curriculum, we have not explored differences in student perceptions between medical universities in East Asia. Follow-up studies using samples of students from different medical universities in East Asia would help mitigate this limitation.
- Fourth, the main purpose of this study was to explore students’ perception toward PT in a qualitative manner. The quantitative part of this study was simply aimed at ensuring that the participants of the focus groups were not extraordinary samples for qualitative analysis. Therefore, the number of participants was quite small for qualitative analysis. Further research will focus more on quantitative analysis using a large sample size within various institutions in East Asia.
- Fifth, we proposed that the application of PT be restricted to later school years, and that the modification of feedback methodology be dependent on school year, according only to students’ perceptions. Opinions and sentiments of other stakeholders (e.g. faculty) are as important as those of students. Thus, it is necessary to evaluate whether this modified PT is acceptable to other stakeholders before implementation.

## Conclusion

This is the first report pertaining to students’ perceptions toward a single pilot progress test in East Asia. The students perceived the concept of PT as suitable for “repetitive memorization”, but as non-suitable to their “narrow approach” learning styles through the end-of-unit tests. Their reliance on post-test explanatory lectures might lessen “confusion” in earlier years, but delay the development of self-directed learning. Key issues in efforts to optimize PT in East Asia may include the administration of PT after the completion of end-of-unit tests and a change in the methods of post-test feedback depending on progress with the curriculum. However, these remarks are based on qualitative analysis of a short period, a small sample-size, and a single-institutional pilot study. The next study should therefore have a longer period, a larger sample size, and be conducted as a multi-center qualitative/quantitative study. Perceptions of other stakeholders should also be investigated in order to explore the optimal use of PT in East Asian institutions.

## Additional files

Additional file 1: Example items of the trial test. (DOC 25 kb) Additional file 2: Table S1. The correct answers of all the items, chosen options in each participant, and the aggregate data in the quantitative analysis. (PDF 240 kb)

## Abbreviations

JMU: Jichi Medical University
PBL: Problem-based learning
PT: Progress testing
