# Supplementary material for: A first report of East Asian students’ perception of progress testing: a focus group study
Source: BMC Med Educ. 2016 Sep 22;16:245. doi: 10.1186/s12909-016-0766-2 (PMC5034519; doi:10.1186/s12909-016-0766-2)
Supplement: Additional file 1: — Example items of the trial test. (DOC 25 kb) [file 12909_2016_766_MOESM1_ESM.doc]

**Example items of the trial test**

**(We used Japanese version for this study. We display English version as a reference.)**

**60. What is the highly specific serological examination item for neuromyelitis optica?**

**A. Anti-ganglioside antibody**

**B. Anti-aquaporin 4 antibody**

**C. Anti-myelin protein zero (P0) antibody**

**D. Anti-myelin basic protein antibody**

**E. Anti-N-methyl-D-aspartate receptor (NMDA) antibody**

**61. A 22-year-old female patient has a severe attack of headache. She had been busy working for 10 days until she began to control it 2 days ago. Yesterday morning, a right temporal headache started when she woke up. The headache deteriorated rapidly and began pulsating. It reached its peak within 30 minutes and she could not keep standing at that moment. The headache got worse when she changed posture or when she walked. After that, the headache gradually ameliorated and diminished after a few hours. She’s experienced the same attacks of headache two to three times per year for three years.**

**The most likely diagnosis is:**

1. **Migraine headache**
2. **Cluster headache**
3. **Tension headache**
4. **Subarachnoid hemorrhage**
5. **Giant cell arteritis**
