# Supplementary material for: A first report of East Asian students’ perception of progress testing: a focus group study
Source: BMC Med Educ. 2016 Sep 22;16:245. doi: 10.1186/s12909-016-0766-2 (PMC5034519; doi:10.1186/s12909-016-0766-2)
Supplement: Additional file 2: Table S1. — The correct answers of all the items, chosen options in each participant, and the aggregate data in the quantitative analysis. (PDF 240 kb) [file 12909_2016_766_MOESM2_ESM.pdf]

		Year 2								Year 3								Year 5							
Item number	*Correct answer*	Male A	Male B	Male C	Male D	Female A	Female B	Female C	Female D	Male A	Male B	Male C	Male D	Female A	Female B	Female C	Female D	Male A	Male B	Male C	Male D	Female A	Female B	Female C	Female D
1	*3*	0	0	0	3	0	3	0	4	0	4	0	0	0	0	5	1	3	3	3	3	3	4	0	3
2	*5*	0	0	0	0	0	0	0	0	0	2	3	0	0	5	5	2	5	5	5	5	5	4	2	5
3	*5*	0	4	0	4	4	4	0	4	4	1	0	0	0	1	1	1	5	5	5	5	0	1	1	5
4	*3*	0	5	0	0	0	0	0	5	0	1	5	3	5	5	4	1	3	3	5	2	3	2	5	3
5	*3*	0	4	3	0	1	3	0	3	3	1	1	1	1	1	1	1	3	1	3	3	3	3	3	3
6	*4*	0	5	0	0	0	2	0	4	1	1	4	0	0	0	1	1	4	4	0	4	4	4	5	4
7	*2*	0	2	0	2	0	0	0	4	0	2	0	0	1	0	0	2	2	2	2	2	2	2	2	0
8	*3*	0	3	0	3	0	3	0	5	3	4	3	0	5	2	3	0	3	3	3	5	3	3	3	3
9	*4*	0	5	0	0	0	0	0	0	0	1	0	0	0	4	0	1	4	4	4	2	0	5	0	4
10	*1*	0	1	0	0	0	4	0	3	1	1	0	0	5	4	1	1	1	1	1	3	1	1	1	1
11	*2*	0	5	0	0	0	5	0	0	1	5	0	0	0	2	2	4	2	2	0	2	2	2	1	2
12	*5*	0	1	0	1	0	2	2	0	0	4	5	0	0	5	5	5	5	1	5	5	5	0	0	1
13	*3*	0	4	0	0	0	0	0	0	1	3	4	4	4	3	3	2	3	3	3	5	4	3	0	3
14	*1*	0	5	0	0	0	3	0	0	0	2	0	0	0	0	0	0	3	1	1	1	1	0	0	1
15	*5*	0	0	0	0	0	5	0	0	2	5	5	0	0	5	5	5	5	3	5	5	5	5	0	5
16	*5*	5	2	4	5	5	3	2	2	1	5	2	0	4	4	5	2	5	5	5	5	1	5	0	2
17	*5*	5	5	1	5	0	5	2	5	5	5	3	5	0	5	5	0	5	5	5	3	5	5	0	5
18	*3*	0	5	0	1	0	1	0	5	4	5	0	0	3	3	3	4	3	3	3	4	3	4	3	3
19	*4*	0	1	0	0	0	5	0	4	4	4	5	3	4	4	4	4	4	4	4	4	4	4	0	4
20	*3*	0	3	0	3	0	5	0	3	3	5	3	3	3	3	3	5	3	5	3	3	0	3	3	3
21	*1*	0	1	0	0	0	0	0	0	5	5	5	5	3	5	5	2	1	1	1	1	2	2	1	1
22	*5*	5	5	1	5	0	5	0	0	5	5	1	0	5	5	0	5	5	5	5	1	5	5	0	5
23	*3*	0	3	3	4	0	1	3	0	3	3	4	0	5	0	1	3	3	3	3	3	3	3	1	3
24	*2*	0	0	0	0	0	4	0	0	0	1	1	0	3	0	0	0	1	2	2	2	0	0	0	2
25	*4*	0	5	2	2	0	5	0	0	0	1	1	4	0	5	3	0	4	4	4	4	4	0	0	5
26	*5*	5	5	5	0	5	2	5	5	5	5	0	5	5	5	2	5	5	5	5	5	4	1	5	5
27	*1*	2	5	0	1	0	3	0	3	3	4	1	0	1	1	1	1	1	1	1	1	0	1	1	1
28	*3*	0	2	0	0	0	3	0	1	3	5	0	2	2	2	0	2	3	3	3	3	3	3	0	3
29	*3*	1	3	3	3	1	1	0	1	3	5	3	3	0	1	5	1	5	1	3	5	3	3	3	3
30	*3*	0	3	0	0	0	2	0	0	3	3	1	0	2	0	1	1	2	3	3	1	0	3	0	3
31	*3*	0	4	4	4	0	0	0	4	0	3	0	0	1	2	0	0	4	3	3	4	3	1	5	3
32	*2*	0	5	0	5	0	4	0	4	2	2	0	0	1	2	2	4	1	2	2	2	1	2	0	4
33	*4*	0	0	3	3	0	0	0	0	0	4	0	0	0	4	3	0	3	4	4	4	4	4	4	4
34	*5*	5	4	0	4	1	2	5	2	5	5	5	5	5	5	5	5	5	5	5	5	5	5	5	5
35	*2*	2	2	2	2	0	2	2	2	2	2	2	1	1	2	2	2	2	2	2	1	2	4	0	2
36	*3*	0	0	0	0	0	0	0	0	1	3	0	4	0	3	1	3	5	3	3	4	3	0	3	3
37	*3*	0	3	0	0	0	0	0	0	1	2	2	4	0	3	3	3	2	3	3	3	1	3	1	3
38	*4*	0	1	2	0	0	0	0	0	0	3	4	0	3	4	0	4	4	4	4	4	4	4	4	4
39	*1*	0	2	0	1	0	0	0	2	0	1	0	0	0	0	1	0	1	1	1	1	0	1	−1	1
40	*5*	0	2	0	0	0	0	0	0	0	5	5	1	1	5	1	5	5	4	5	5	5	5	5	5
41	*3*	0	3	4	0	0	0	0	3	3	4	2	3	1	3	3	3	3	3	3	3	3	3	2	3
42	*2*	0	0	0	0	0	2	0	3	2	1	0	0	0	1	2	0	2	2	2	2	1	2	0	2
43	*4*	0	1	4	3	0	4	0	5	4	4	4	2	4	4	2	4	4	4	4	4	4	4	1	4
44	*1*	0	4	0	0	0	5	0	5	0	4	0	0	0	1	0	0	2	1	0	2	0	0	4	1
45	*4*	0	2	0	0	0	0	0	0	3	5	0	0	5	4	4	4	4	4	4	4	4	4	4	4
46	*2*	0	2	2	3	0	2	2	3	4	2	0	0	2	2	2	3	2	2	2	2	0	2	0	2
47	*1*	0	0	0	0	0	3	0	0	0	1	3	1	0	2	3	2	1	1	1	1	1	1	1	1
48	*3*	0	0	0	0	0	0	0	2	4	4	0	0	0	0	5	0	3	3	3	3	3	3	3	3
49	*5*	0	1	0	0	0	0	0	4	5	5	0	0	0	5	5	0	5	5	5	5	5	5	5	5
50	*1*	0	0	0	0	0	0	0	1	0	4	0	2	0	1	0	0	1	1	1	1	1	5	1	1
51	*1*	0	0	0	0	0	0	0	4	1	1	1	1	0	2	4	1	1	1	2	1	0	1	0	1
52	*1*	0	0	0	0	0	0	0	0	2	1	4	0	4	1	2	1	1	1	1	2	1	1	1	1
53	*1*	1	1	1	1	1	1	1	3	1	1	1	1	1	1	1	1	1	1	1	5	5	1	1	5
54	*2*	0	0	0	0	0	0	0	2	2	2	2	0	0	2	2	2	2	2	2	2	5	2	3	2
55	*5*	0	5	0	0	0	0	0	1	0	0	0	0	0	5	0	5	5	5	5	3	5	5	5	5
56	*3*	0	0	0	0	0	0	0	3	3	3	5	3	0	3	3	0	3	3	3	3	3	0	−1	3
57	*2*	0	3	0	4	0	2	2	2	2	3	1	1	2	2	2	3	3	3	2	2	0	2	1	2
58	*3*	0	0	0	0	0	0	0	0	4	0	1	0	0	3	3	3	3	3	3	2	3	3	0	3
59	*3*	0	0	3	0	0	0	0	0	2	2	3	1	0	3	2	3	3	3	3	4	3	4	0	3
60	*2*	0	0	0	0	0	0	0	1	2	4	0	0	2	2	2	2	2	2	2	2	2	2	2	2
61	*1*	0	2	0	1	0	5	0	1	1	1	1	1	1	1	1	1	1	1	1	1	1	1	0	1
62	*3*	3	3	0	3	0	3	0	4	3	3	5	1	3	3	3	3	3	3	3	3	3	3	3	3
63	*5*	0	0	0	5	0	2	0	0	5	5	0	1	4	5	5	5	5	5	5	5	5	5	5	5
64	*1*	0	1	1	1	1	1	0	0	1	1	1	0	0	0	1	1	1	1	1	1	1	1	1	1
65	*4*	0	3	0	4	0	5	5	0	4	5	0	5	0	4	3	4	4	4	4	4	4	4	0	4
66	*5*	5	3	5	5	5	5	0	5	5	5	5	0	5	5	5	5	5	5	5	5	5	5	5	5
67	*4*	0	0	4	0	4	0	0	4	4	4	0	2	2	1	4	4	4	4	4	4	4	4	0	4
68	*2*	0	4	0	4	0	1	0	0	2	2	4	4	2	2	3	2	4	2	2	2	2	2	2	2
69	*5*	0	4	0	5	0	0	0	0	5	5	0	5	5	5	5	5	5	5	5	5	5	5	0	5
70	*5*	5	2	5	4	0	3	3	2	0	5	5	5	0	5	4	2	5	5	5	5	0	4	0	5
71	*5*	0	0	0	0	0	0	0	0	4	2	0	0	5	3	3	0	5	5	5	5	5	5	5	5

72	3	0	2	3	0	0	0	0	0	3	3	0	3	3	3	3	3	2	3	3	1	3	3	2	3
73	1	0	1	1	5	1	1	1	1	1	1	1	1	1	0	1	1	1	4	1	1	5	5	1	5
74	4	0	3	0	3	4	3	0	0	4	4	4	4	3	4	4	5	4	4	4	3	4	4	4	4
75	2	1	2	0	2	0	1	0	2	0	2	0	0	0	2	2	2	2	2	2	0	2	1	2	2
76	5	5	5	5	5	0	5	0	4	5	5	5	5	5	5	5	5	5	5	5	5	5	5	5	5
77	3	0	3	0	4	0	4	0	0	4	4	4	5	4	3	3	3	3	3	3	1	3	3	3	3
78	5	0	5	3	3	0	4	0	2	0	0	5	0	5	0	5	2	5	5	0	2	5	5	5	5
79	2	0	5	0	0	2	0	0	0	2	4	0	3	5	4	5	1	2	2	2	2	2	5	2	2
80	4	0	5	0	3	0	3	0	2	0	5	3	1	0	4	4	0	1	4	4	2	3	4	4	4
81	5	5	4	0	5	5	5	2	1	5	5	5	1	5	5	5	5	5	5	5	4	5	1	5	5
82	1	0	0	0	0	2	0	0	0	3	3	0	0	2	3	5	5	1	1	1	1	1	1	0	1
83	5	0	0	0	0	0	0	0	0	0	4	0	0	4	0	4	4	0	5	5	4	1	1	0	1
84	2	0	0	0	0	0	0	0	0	0	4	0	0	0	0	0	0	0	2	2	1	4	0	3	2
85	2	0	5	0	5	0	0	0	5	1	5	2	0	2	2	2	5	2	2	2	2	2	2	0	2
86	1	0	4	0	0	0	0	0	0	0	2	2	1	0	1	1	2	1	5	0	5	0	1	0	5
87	1	0	5	0	0	0	5	0	0	1	1	0	0	0	2	1	1	1	2	5	1	3	5	0	1
88	4	0	2	3	0	0	2	0	0	0	2	3	0	0	0	4	0	4	4	2	2	4	3	0	3
89	1	0	5	0	0	0	0	0	0	0	0	0	0	0	0	5	1	1	1	1	3	3	0	0	1
90	2	0	0	0	0	0	0	0	0	0	0	0	0	0	0	0	5	2	2	2	4	2	0	0	2
91	2	0	0	0	3	0	2	0	0	0	2	0	0	0	2	2	0	2	2	5	4	2	2	0	2
92	2	0	0	0	0	0	5	0	0	0	4	0	3	0	4	2	2	2	2	2	2	4	3	0	2
93	1	0	1	0	1	0	0	0	1	1	4	1	1	5	1	1	1	1	1	1	1	1	1	1	1
94	1	0	0	0	0	0	1	0	0	4	5	5	1	1	1	1	1	1	1	1	1	1	1	1	1
95	5	0	4	4	3	0	1	0	5	5	4	5	0	0	5	2	1	5	5	5	5	5	2	5	5
96	5	0	3	0	0	0	0	0	0	5	4	5	5	0	5	5	0	5	5	5	5	5	5	0	5
97	4	0	3	0	0	0	0	0	0	0	3	0	0	5	4	4	4	4	4	4	4	4	4	0	4
98	2	2	2	2	2	2	2	0	2	2	2	2	4	2	4	2	2	2	2	2	2	2	2	0	2
99	1	0	4	0	0	0	0	0	0	1	2	2	1	1	1	1	1	1	1	1	1	1	1	0	1
100	5	0	0	0	0	0	0	0	0	5	5	0	5	0	5	5	5	5	5	5	5	3	5	4	5
Correct (%)		13	26	17	24	11	22	8	20	47	47	29	25	27	62	58	50	84	90	90	67	70	67	42	90
Incorrect (%)		3	46	11	22	5	35	6	32	22	48	29	24	29	21	29	30	14	10	5	32	17	23	18	9
"Don't know" (%)		84	28	72	54	84	43	86	48	31	5	42	51	44	17	13	20	2	0	5	1	13	10	40	1
Total		100	100	100	100	100	100	100	100	100	100	100	100	100	100	100	100	100	100	100	100	100	100	100	100
Formula score		12.25	14.50	14.25	18.50	9.75	13.25	6.50	12.00	41.50	35.00	21.75	19.00	19.75	56.75	50.75	42.50	80.50	87.50	88.75	59.00	65.75	61.25	37.50	87.75

Y2 n=8	Mean	SD
Correct (%)	17.63	6.48
Incorrect (%)	20.00	16.21
"Don't know" (%)	62.38	22.10
Formula score	12.63	3.53

Y3 n=8	Mean	SD
Correct (%)	43.13	14.36
Incorrect (%)	29.00	8.45
"Don't know" (%)	27.88	16.60
Formula score	35.88	14.53

Y5 n=8	Mean	SD
Correct (%)	75.00	16.89
Incorrect (%)	16.00	8.62
"Don't know" (%)	9.00	13.37
Formula score	71.00	18.31
